# Expanding access to genomic analysis and reporting in research studies: The GENYSIS research core

**DOI:** 10.1017/cts.2026.10696

**Published:** 2026-02-09

**Authors:** Kimberly S. Foss, Tam P. Sneddon, Eleanor P. Fensterle, Scott A. Melville, Mai Xiong, Christopher W. Gregory, Paul Greenwood, Kelly A. Rafferty, Elizabeth K. Hutchins, Neeta L. Vora, Kelly L. Gilmore, Senyene E. Hunter, Karen E. Weck, Jonathan S. Berg, Bradford C. Powell

**Affiliations:** 1 Department of Genetics, The University of North Carolina at Chapel Hill, USA; 2 Department of Pathology and Laboratory Medicine, The University of North Carolina at Chapel Hill, USA; 3 Department of OB-Gyn, The University of North Carolina at Chapel Hill, USA; 4 Department of Neurology, The University of North Carolina at Chapel Hill, USA

**Keywords:** Shared research resource, core facility, expanding access to genetics, genetics research, genetic testing

## Abstract

**Introduction:**

The growing utility and availability of genome-scale sequencing has led to increasingly broad incorporation across specialty disciplines of clinical research. However, classification of clinical relevance of genetic variation is an inherently clinical task, and the expertise to perform the necessary analysis, confirmation and reporting of these variants is not available in all research teams; consequently, disclosure of genomic variants to research participants remains challenging for many researchers. Advancing genomic medicine as a standard of care first requires institutional commitment and partnerships in supporting genomics in varied research studies that are inclusive of return of results to participants.

**Materials and methods:**

The University of North Carolina at Chapel Hill has vast experience with genetics in both clinical and research realms. By utilizing historical experience and input from key players, the Clinical GENomic analYSIS (GENYSIS) core facility was created as a case study and aims to provide a roadmap for research organizations to implement their own genomic sequencing core facilities.

**Results:**

The core has established a molecular sign-out conference, partnered with other core facilities on campus, and provides five main services: bioinformatics, variant analysis, clinical reporting, post-test services, and consultation with project advising. This paper presents case examples with discussion of continuous methodology improvements and embedded educational activities.

**Conclusion:**

This novel shared research resource enables clinical researchers with limited staff and genomics expertise to provide clinically relevant results to their study participants, expanding the reach of genomics research.

## Introduction

Genomic sequencing technologies, particularly exome and genome sequencing (ES/GS), are increasingly integrated into both clinical and research settings due to their power to reveal the genetic basis of disease and deliver diagnostic clarity [1]. Genome-scale sequencing is now recommended as standard of care for multiple clinical indications [2–5] with utility for other indications actively under evaluation. As with many transformative technologies, genomic sequencing has expanded beyond the confines of clinical genetics into diverse domains of medical research.

To fully realize the potential of genomic technologies in improving human health, it is essential that ES/GS be incorporated into research led by investigators across a range of specialties who may not have formal training in medical genetics. However, effective implementation of ES/GS in clinical research involves more than just sequencing [6]. The combined tasks of weighing evidence about the possible effect of genetic variants, determining pathogenicity, and returning clinically meaningful results to participants – especially in a form suitable for integration into clinical care – require a multidisciplinary infrastructure, including clinical geneticists, laboratory geneticists, variant scientists, bioinformaticians, and genetic counselors. Most research studies do not have the capacity or funding to build and sustain such a vast team independently.

In addition, returning secondary findings has become a routine component of many ES/GS pipelines and is recommended by the American College of Medical Genetics and Genomics [7]; however, researchers may lack the necessary experience or infrastructure to incorporate this offering effectively into their study protocols [8]. While commercial laboratories can support some aspects of sequencing and variant analysis, they often offer less flexibility or the collaboration needed for many research protocols, particularly those requiring custom analytic approaches or involving participants who have previously had negative clinical genetic testing. Furthermore, the capacity for nuanced phenotypic reanalysis and iterative communication with research teams is often limited in commercial settings.

These challenges are compounded by the evolving ethical and regulatory landscape surrounding the return of genomic research results. In the early years following the release of the first draft of the human genome, most institutional and regulatory frameworks restricted or outright prohibited the return of individual research results to participants. This cautious approach reflected concerns about analytic validity, clinical utility, additional costs that could be incurred by research teams, and potential for misinterpretation of findings in the absence of clinical context. However, dialog among researchers, bioethicists, clinicians, and research participant advocates led to a substantial shift in expectations [9,10]. There is now growing consensus that clinically relevant primary and secondary findings should be made available to research participants when accompanied by appropriate informed consent and validation of those results (e.g., within CLIA-certified laboratories) as this information can be used for downstream medical management and care. This shift in perspective underscores the importance of integrating rigorous clinical analysis and ethical return-of-results processes into genomic research studies. Although it has been proposed that research institutions develop centralized services to provide secondary genomic findings to research participants [7,8,11–14], we are unaware of any genomics research core facilities that go beyond laboratory and bioinformatics support for genomic sequencing technologies and provide comprehensive clinical genomic analysis services, including customizable bioinformatic pipelines, in-depth variant curation, discussion of variants with a multidisciplinary team, and clinical confirmation of suspicious results, along with results disclosure including a report that can be added to the patient medical record.

Over the past decade, researchers at the University of North Carolina at Chapel Hill (UNC-CH) have built a multidisciplinary team with expertise in clinical genomic research, including the identification and return of actionable genetic findings. To support broader access to this infrastructure, the UNC-CH Program for Precision Medicine in Health Care (PPMH) established the Clinical GENomic analYSIS core research facility – a shared research resource aimed at providing end-to-end genomic services for research studies led by investigators without dedicated genetics expertise. As a service-based core facility, GENYSIS allows investigators to budget for comprehensive clinical analysis as a defined line item, avoiding the need to support effort for individual expert personnel. This shared resource model represents a scalable, interdisciplinary approach to clinical genomic research and enables clinical researchers with limited staff and genomics expertise to return clinically relevant results to their study participants, expanding the reach of genomics research.

Here we present the establishment of the GENYSIS core facility at our institution. Our implementation methods involved engagement with prospective clients and other institutional stakeholders to define the key components and rate structures for a sustainable facility. The proximal products of this implementation research include the established production service processes and the lessons learned in facility creation. To demonstrate expansion of diagnostic capability for studies without internal clinical genetics expertise, we describe exemplar cases with discussion of benefits for the research community.

## Materials and methods

### Identification of key components of the service

UNC-CH has an extensive history of both clinical and research genetics. Several research projects demonstrate the past and continued ability to combine research analysis and clinical reporting at our institution (see Table 1). In initial planning for the GENYSIS core facility, key stakeholders from these projects were engaged to identify the foundational lessons from each, noting factors that would likely be of importance to future research clients’ studies. These discussions expanded to utilization of emerging tools for genomic variation analysis that researchers would like to incorporate in future studies.

**Table 1.** UNC research studies that informed the development of the GENYSIS research core, along with their description, project duration, number of completed cases, and main foundational lesson. While previous projects varied in aims and structure, all helped guide the emerging process by which the multidisciplinary research team reviewed genetic variants and discussed which variants should be returned to the patient. As with other genomic sequencing studies, many of these research projects also included the return of secondary findings, as was becoming more common when performing exome and genome sequencing [7]

Prior studies	Description	Project duration	#Cases	Foundational lessons informing GENYSIS implementation
NCGENES	Pediatric and adult proband exomes in a diagnostic context	2012–2016	631	Customization of variant analysis and reporting, phenotype-based prioritization [14,15], multidisciplinary discussion of results, reporting of secondary findings [16,17] and communication of uncertainty [16,18].
GeneScreen [13,14]	Actionable finding gene panels in a population screening context	2013–2018	264	Adaptations of analysis and reporting for participants with low pre-test probability of clinically-relevant results.
Fetal exome study [15]	Fetal trio exome in a diagnostic context	2014–2019	102	Trio analysis; Prioritization of variant analysis for phenotypes that cannot be well-defined or for which there is not substantial prior literature.
NC NEXUS [16]	Exome sequencing as a newborn screening modality	2017–2019	106	Customization of analysis to screening vs. affected cohort; with initial blinding to cohort followed by additional analysis in affected cohort to evaluate sensitivity
NCGENES 2 [17]	Pediatric proband exomes in a diagnostic context	2018–2021	101	Classification of case-level results based on clinical phenotype, as modified by subsequent phenotyping
PrenatalSEQ study (part of multi-site study)	Fetal trio genomes in a diagnostic context	2019–2025	149	Importance of development of infrastructure for cross-institution collaboration [19]
Fetal brain anomaly (FBA) and anencephaly studies	Fetal trio exomes/genome in a diagnostic context	2021– present	>100 with ongoing enrollment	Prioritization of variants identified in human subject for which evaluation in a model organism may clarify classification [20]

To mature the lessons learned into a shared research resource requires understanding how different business and operational models function for sustainability. Shared research resources (core facilities) offer access to subject matter experts, high-end instrumentation, and standardized experimental practices at a competitive price and are essential to the success of funded research programs at academic institutions. Representing a wide array of research domains and service offerings, core facilities also enhance scientific collaboration across institutions. Based on these key attributes and the obvious need for more robust analysis of genetic variants, we determined that GENYSIS should be established as a shared research resource at UNC-CH.

The application process for GENYSIS core facility approval began in 2021 with the preparation of an institutionally required operating plan and justification of proposed rates. The core facility was housed jointly within the Department of Pathology and Laboratory Medicine and the Department of Genetics, where GENYSIS leadership hold appointments. The GENYSIS Director is a clinical laboratory geneticist, the Faculty Director is a medical geneticist, and the Assistant Director is a genetic counselor. The chairs of both departments were engaged throughout the planning process, and both approved the operating plan prior to submission for approval at the university level.

### Defining itemized services and rates

The UNC-CH institutional policy on rate structure requires that core facilities only charge for services with rates approved by the Office of Sponsored Programs (OSP) and rates charged to internal clients (investigators within the UNC system) are set at breakeven, where revenue equals expenses. One goal in planning for specific billable rates was to provide modularity to support studies with varied research designs, mirroring the studies in Table 1. To achieve this, we considered which services might be independently utilized by only some studies.

The charges for bioinformatic analysis, variant classification, and clinical reporting were created by considering the rates offered by clinical laboratories and internal charges for confirmatory Sanger sequencing or qPCR analysis in a CLIA-certified laboratory, and by averaging the estimated volume, complexity, and time needed to analyze each case. The rate for custom analysis (partitioned hourly) was based on the proportion of effort of a bioinformatician. The rate for post-test services was estimated based on billing codes utilized for clinical genetic counseling services at the time (Current Procedural Terminology (CPT) code 96040). A separate hourly rate for general consultation with the study team was made available to allow further flexibility as needed for additional requirements of expertise of core directors, while one free consultation is available per project to solidify GENYSIS services are an appropriate fit. The core facility was approved in early 2023, with rates expected to be reviewed and adjusted at least every two years.

## Results

### Incorporation of key common features of prior studies

Discussions with lead investigators of prior studies and prospective clients revealed both baseline characteristics that are required for any analysis of clinical genetic information (e.g., standardization of analysis and reporting), as well as features that could be seen as key approaches or differentiating factors between an academic shared research resource when compared to commercially-available testing (e.g., embedding of educational activities and capacity to evaluate emerging technologies). These factors are summarized in Box 1.

Box 1: Key facilitators and strategic approaches for a clinical genomic analysis shared research resource:

- Standardization of analysis and reporting with automated techniques for primary sequence analysis with meticulous record of analysis provenance, and personnel with appropriate training and expertise to provide clinical classification of sequence and structural variants.
- Leveraging existing institutional resources.
- Multidisciplinary discussion of sequence results, ideally including clinical geneticists, other medical specialists as needed, laboratory geneticists, genetic counselors, and variant scientists (i.e. Molecular Sign-Out meeting).
- Post-test services to communicate the results to participants and/or healthcare providers, and identification of appropriate resources to aid understanding and utilization of clinical results.
- Embedded educational activities, such as training of primary variant scientists, participation in sign-out discussions, and inclusion in development of custom informatic analyses, incorporating trainees from client studies into the sign-out conference.
- Continuous improvement: Capacity to evaluate emerging analytic techniques or sequencing technologies, and for validation of laboratory-developed tests in a CLIA environment.
- Engagement with the global genomic medicine community to improve the ability to solve rare disease cases.
- Contribution to the delineation of newly described syndromes.

Investigators and institutional stakeholders also recommended that the facility focus on aspects that could be uniquely provided by the core facility and were not duplicative of other available facilities. Previous studies had utilized other research cores, such as the UNC BioSpecimen Processing (BSP) facility for DNA extraction and banking, as well as the UNC High Throughput Sequencing Facility (HTSF) for ES/GS. Investigators also requested flexibility regarding how samples or genetic data could enter the GENYSIS analysis process. This desire for flexibility was incorporated with modifications to our informatics pipeline to allow procedures to start from unaligned reads, aligned reads, or previously called variants.

A multidisciplinary Molecular Sign-Out conference, including research study investigators, clinical geneticists, laboratory geneticists, bioinformaticians, variant scientists, and genetic counselors, has been convened during the course of UNC’s early genomic research projects and met weekly for over a decade. These meetings were designed to present analysis results and foster discussion of the potential significance of variants with review of evidence from the literature, genetic databases, and phenotype/genotype correlation. The ability of study investigators to participate was identified as a key differentiating factor. As part of the UNC-CH academic medical center, institutional stakeholders also emphasized that it is important for the GENYSIS core to provide educational opportunities for students at varying levels in their careers. The Molecular Sign-Out meeting has been a rich learning opportunity for a variety of trainees, including 17 undergraduate students [15], 7 post- baccalaureate interns and research coordinators, 4 graduate students, 5 medical students, and 5 postdoctoral/medical residents/fellows. In addition, medical students have participated in data review and case analysis as part of elective projects. Genetic counseling graduate students have worked on variant curation during research rotations and the core has hired a post-baccalaureate intern to gain experience in the field for ongoing professional development. In addition, part of the Carolina Health Informatics Program Master’s degrees at UNC requires a professional internship and the core has worked continuously with one of these graduate students.

### Services

**Figure 1.**
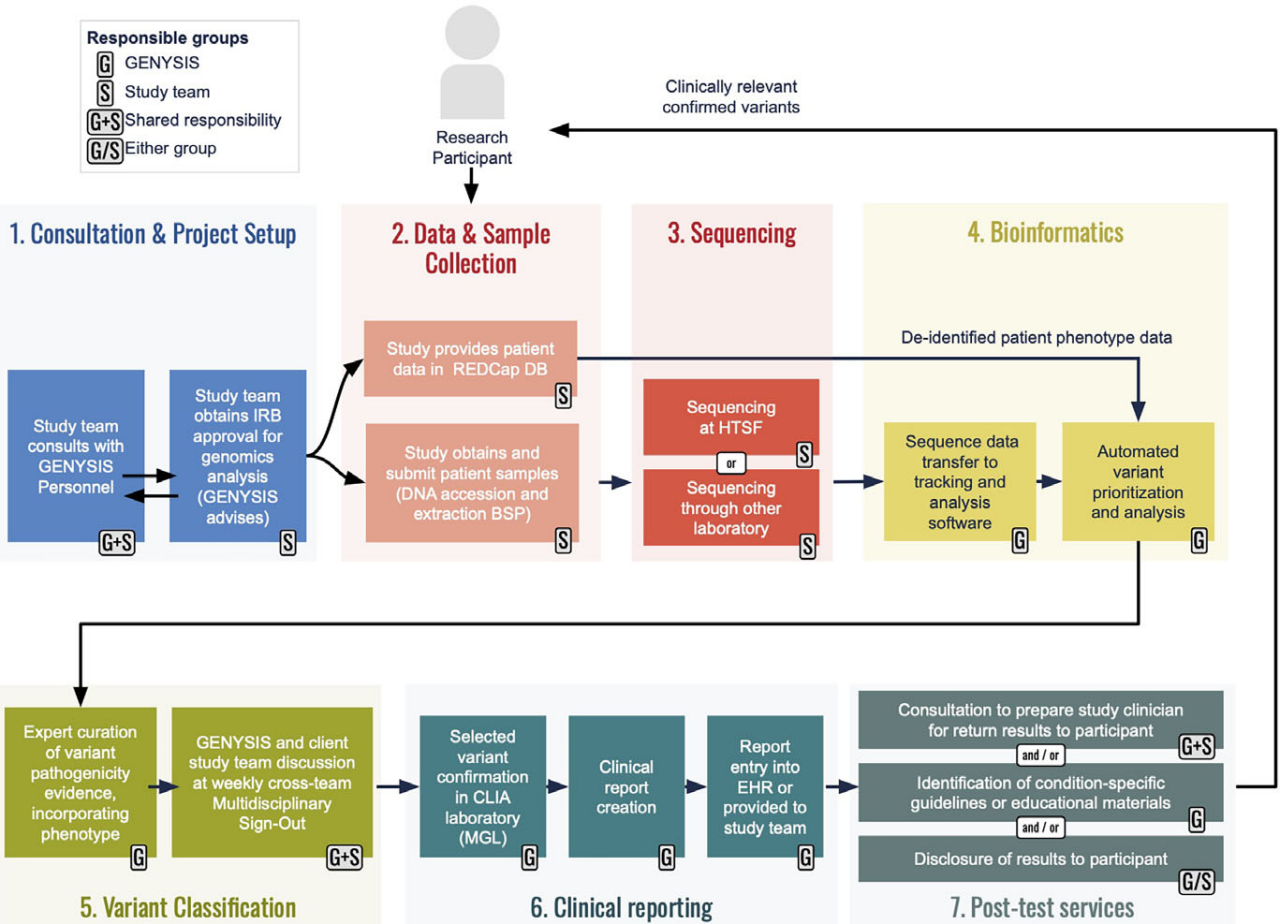
An overview of workflows for GENYSIS-supported projects. Prospective client researchers are encouraged to meet with GENYSIS leadership (1) early in their planning so we can help identify relevant services and provide input on Instituational Review Board (IRB) protocol preparation. The studies are responsible for sample collection/accessioning and for phenotypic data collecton (2), with many using UNC’s existing Biospecimen Processing (BSP) facility and secure RedCap database, respectively. Studies may have sequencing (3) through the UNC High Throughput Sequencing Facility (HTSF) or elsewhere. GENYSIS becomes directly involved again at the time of bioinformatic analysis (4) which we can initiate from sequence reads (e.g., FASTQ or BAM files) or partially analyzed data (e.g., variant call files), depending on study design. Our analysis incorporates study-provided phenotype into in-house developed annotation and prioritization pipelines. Following prelimiary curation and classification from a GENYSIS variant scientist, personnel from client studies are encouraged to participate in our multidisciplinary conference for discussion of potentially reportable findings (5). Clinical reporting (6) includes Sanger sequencing or qPCR confirmation in the College of American Pathologists (CAP)-accredited, CLIA-certified Molecular Genetics Laboratory to produce a clinical laboratory report that can be included in the participant’s electronic medical record. GENYSIS offers an array of post-test services (7) to ensure that studies can responsibly return clinically relevant research results to participants.

The GENYSIS core facility provides five main services: bioinformatics, variant analysis, clinical reporting, post-test services, and consultation and project advising (see Figure 1).

1. **Bioinformatics** – the group uses well-established tools, providing the analytic stability expected from a clinical laboratory. Automation is coordinated in a high-performance computing environment using Slurm [16] for queuing and resource management and Snakemake [17] to coordinate tasks and parallelization. The primary pipeline includes alignment (bwa-mem), marking of duplicates (Picard Mark Duplicates), variant calling (FreeBayes) and automated variant annotation with reference data from external sources including ClinVar and gnomAD. Metadata of program and reference data versions are recorded with each sample run. Versions of this pipeline have been used in previous projects mentioned in Table 1, and the pipeline was a portion of the investigational device [18]
  For projects that require additional analysis beyond what is available in the standard pipeline, staff bioinformatics support is available on a time unit basis. The standardized bioinformatic analysis (starting from unaligned or aligned sequence reads or from previously called variants) was included in the rate structure, but we also provide capacity for additional customization. To make the analysis service available to as many researchers as possible, initial sequencing was not required to be performed in a CAP-accredited/CLIA-certified environment. This has implications for the potential sensitivity of detection of variants, which we mitigate through using permissive variant calling thresholds for evaluation of potential causative variants. We also incorporate manual review of read data around regions of specific interest (e.g., when a single pathogenic variant is found in a gene associated with a recessive condition that could explain a participant’s phenotype).
2. **Variant Analysis** – Variants are loaded into in-house software for filtering and curation by variant scientists, whom are trained in-house by our Laboratory Genetics and Genomics (LGG)-certified director using an internal standard operating procedure, along with resources from the ClinGen Biocurator Core at UNC. The variant analysis workflow is consistent with the Clinical Genomic Resource (ClinGen) best practices and synthesizes data from various public databases such as the Allele Registry, ClinVar, gnomAD, and OMIM to find support for any variant specific pathogenicity. Variant classification is based on American College of Medical Genetics and Genomics (ACMG)/Association for Molecular Pathology (AMP) guidelines [19]. Variants are curated with de-identified phenotypic information provided by the research study. Supplementary literature findings via sources such as PubMed, and Gene Reviews, along with others, are then used to find additional genotype-phenotype correlations based on the proband’s presentation. Secondary findings are reportable following the ACMG guidelines [20].
  For analysis of genetic data, the shared research resource offers ACMG secondary findings analysis only, ≤ 200 gene panel analysis, or >200 gene panel/exome/genome short-read or long-read analysis with or without secondary findings (depending on participant consent), recognizing the differences in complexity of analysis at these different scales.
3. **Clinical Reporting** – Clinical confirmation of a research result in proband and comparator samples is offered to be accompanied by a clinical laboratory test report for the proband. Following orthogonal Sanger or qPCR confirmation in the UNC Health CLIA-certified Molecular Genetics Laboratory (MGL), reports are written for variants that meet the reporting criteria of the study. Reports are written for likely pathogenic and/or pathogenic variants in conditions related to patient phenotypic features (primary findings). In addition, Variants of Uncertain Significance (VUS) for primary conditions may be included in clinical reports if there is sufficient phenotypic overlap as determined through multidisciplinary discussion and when consistent with project-specific study design and IRB requirements. Reports are written by a genetic counselor and/or clinical laboratory geneticist after variants are presented at Molecular Sign-Out conference and approved by the research project’s Principal Investigator. Reports are reviewed and signed by a laboratory geneticist. Secondary and/or incidental findings are reported if included on the project-specific IRB-approved consent form and the proband and/or their guardian has consented to these specific findings. Clinical reports are issued to the research team via secure shared storage in RedCap and can be added to the patient’s UNC Health electronic medical record. Because only positive results would be confirmed clinically, clinical reports are only available for those results; negative research reports are not typically produced.
4. **Post-test services** – Assistance with post-test result disclosure is available for all projects, although may not be needed for investigators with clinical genetics expertise. These services can include discussing the results with the research team, reviewing educational materials that may assist with result disclosure, and offering guidance on condition-specific resources. Alternatively, a genetic counselor can directly contact the research participant with documentation of this disclosure, if approved by the project’s IRB. Clinical genetic counseling is always encouraged following each result disclosure.
5. **Consultation and Project Advising** – GENYSIS provides a free 1-hour virtual or in-person consultation for any interested clinical genomic researchers. This may include scheduling joint meetings with BSP and/or HTSF staff to help with imminent project coordination. During our initial consultation on projects, the GENYSIS team works with client investigators to estimate factors that may impact budgeting for their project (i.e. estimating the number of positive and inconclusive results that would require Sanger sequence confirmation based on cohort characteristics and expected diagnostic yield). Conversations may also include reviewing language and protocols included in informed consent documents, including discussion around secondary and incidental findings. Additional consultations and advising are available on an as-needed basis.

### GENYSIS contributions to research studies

To date, GENYSIS has worked with investigators on several projects from varied clinical domains, including diagnostic studies seeking genetic etiology for neurodevelopmental disorders (NDDs), auditory neuropathy spectrum disorders, schizophrenia, chronic kidney disease, and hereditary breast cancer. These studies would not have been able to have genetic variants evaluated and returned to research participants without the services provided by GENYSIS, showing increase in reach of genetic research at our institution. In addition, the GENYSIS team has held free virtual consultations with investigators interested in analyzing genome data from patients with pediatric cancer, nephrotic syndrome, immunodeficiency, Parkinsonism, primary ciliary dyskinesia, cystic fibrosis, and repeat expansion disorders, in addition to clinical re-analysis, pharmacogenomics, and population age-based genomic screening.

**Figure 2.**
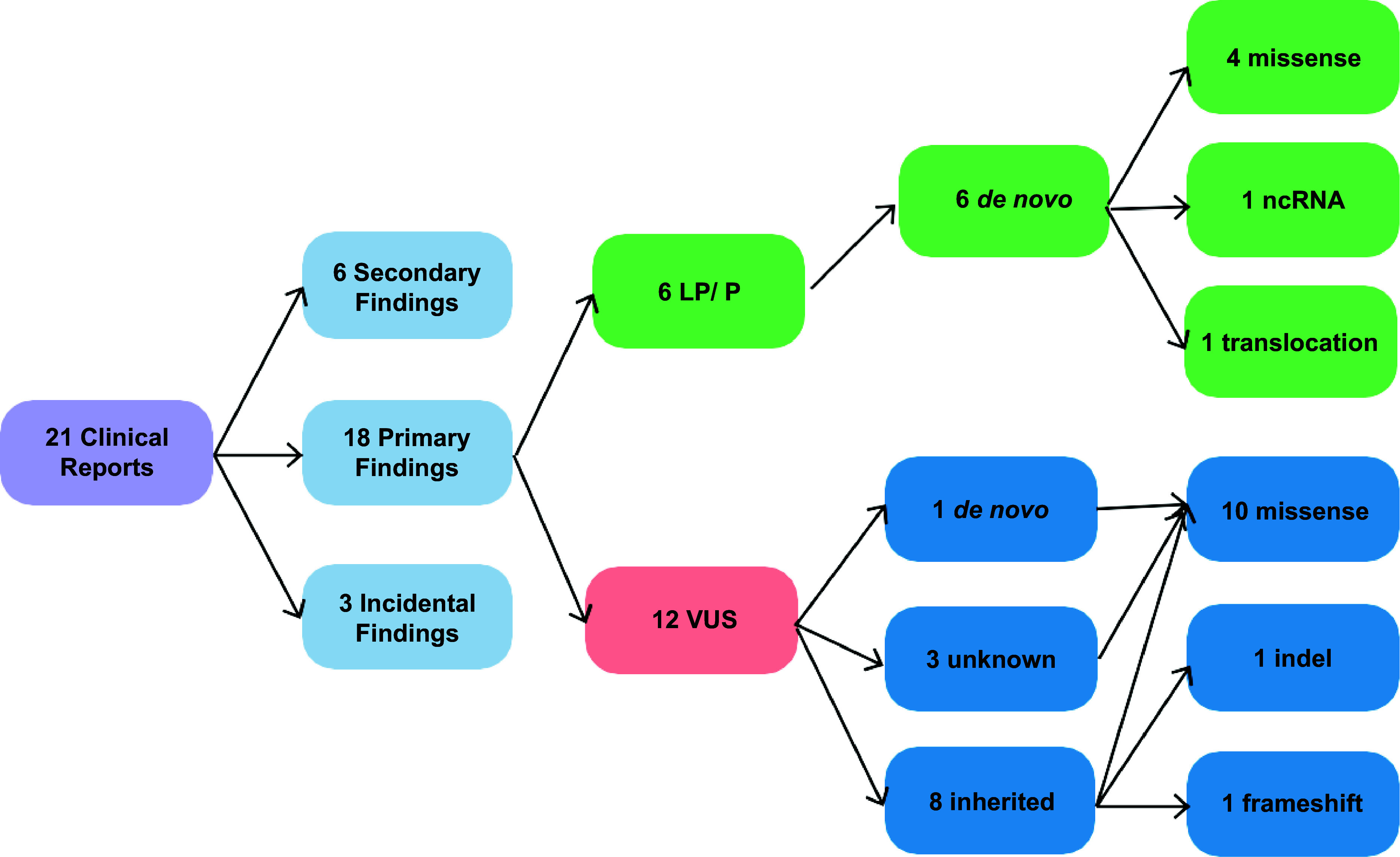
Breakdown of the 27 variants reported in the 21 clinical reports released in the electronic medical record by GENYSIS.

Findings returned to participants and reported through the medical record include novel sequence and structural variants, ACMG recommended secondary findings, and incidental findings deemed actionable by the interdisciplinary GENYSIS Molecular Sign-Out team. Within the studies consented for return of results, between February 2023 and July 2025, there were 118 participants with exomes or genomes analyzed by GENYSIS. Most were proband only, with 28 cases being trios and 7 cases being duos. We issued 21 clinical reports (17.8% of cases) consisting of 27 variants (see Figure 2). Again, no clinical report is produced for a negative analysis. Thirteen of the reports included variants that were causative or suspicious to be causative of the primary indication for testing (11.0% of cases). Of these 13 reports, 6 included pathogenic or likely pathogenic variants. In addition, 6 ACMG secondary findings were reported (5.1% of cases). Three incidental findings were reported, defined as non-diagnostic of the primary indication, yet remain actionable and are not in genes included on the ACMG secondary findings list (2.5% of cases). Demographic information for participants will not be reported, as collaborating research projects have proprietary use of this information. See Figure 2 for additional details.

Of the 18 primary findings, six included a *de novo* pathogenic/likely pathogenic (P/LP) variant in a gene that was not on previously tested clinical gene panels. The remaining 12 variants were classified as uncertain significance. One of these variants was *de novo* and correlated with recent literature but had limited gene-disease validity. The remaining 11 VUS results were related to the patients’ reported phenotypes. Reportable variant details are added to Epic as discrete values to facilitate future identification and, if consented, are submitted to ClinVar (Organization ID: 509948). See Box 2 for illustrative case examples.

Box 2: GENYSIS case examples illustrating benefits of the shared research resource. Full details are not presented here, please see published references for additional details.

- **Case 1:**
  - **Finding:** A single-exon deletion in *PEX1* was identified in an infant.
  - **Impact:** Accounted for a second allele that was missed on a clinical panel [26, 27].
  - **Role of GENYSIS:** Illustrates the use of emerging analytic technologies as requested by client investigators, in this case for detection of structural variants and copy number variants.
- **Case 2:**
  - **Finding:** A complex translocation-inversion involving the *ARID1B* gene.
  - **Impact:** Resolved using the Oxford Nanopore long-read sequencing analysis pipeline [28, 29]. We performed sequencing across breakpoints to confirm the chromosome rearrangement.
  - **Role of GENYSIS:** Illustrates the use of emerging analytic technologies as requested by client investigators.
- **Case 3:**
  - **Finding:** A highly suspicious *GLUL* gene variant classified as a variant of uncertain significance due to limited gene-disease validity for an autosomal dominant disorder.
  - **Impact:** Study investigators were provided additional information about ongoing research regarding this gene. A GENYSIS variant scientists, who also works with ClinGen, collaborated with the Epilepsy GCEP [30] to curate *GLUL* as having Moderate evidence for developmental and epileptic encephalopathy [31]. This participant’s variant was also submitted to the GeneMatcher database, with the participant now in the process of being recruited for functional studies at a separate institution to further investigate the gene-disease association.
  - **Role of GENYSIS:** Illustrates the benefit of a large research network and facilitating access to additional research.
- **Case 4:**
  - **Finding:** A research participant with an *AP2S1* variant of uncertain significance reported on exome analysis by a commercial genetic testing lab that previously closed, thus preventing re-analysis by the original lab.
  - **Impact:** At the time of exome analysis, *AP2S1* only had a disease association with hypocalciuric hypercalcemia, type III [32], which was not considered a phenotypic match with the participant. Upon re-analysis by GENYSIS, a cohort of individuals with *AP2S1* variants was identified in a pre-print publication associated with a neurodevelopmental disorder (NDD) phenotype [11,33].
  - **Role of GENYSIS:** Illustrating how GENYSIS can facilitate access to additional research, the lead author of the preprint was contacted, and the participant is now included in their research cohort. Once the full characterization of that cohort is published, we plan to use the additional cases as evidence to support the gene-disease relationship, potentially upgrading the VUS to likely pathogenic, and issue an updated clinical report to the patient’s medical record.
- **Case 5:**
  - **Finding:** A variant of uncertain significance was detected in the *SCAF8* gene, for which a clear gene-disease relationship has not been established. However, the *SCAF4* gene is associated with an autosomal dominant neurodevelopmental disorder, and the *SCAF4* and *SCAF8* genes are paralogs, thought to have arisen via a gene duplication that occurred in vertebrates [34].
  - **Impact:** Functional studies have demonstrated that the lethal *SCAF4/SCAF8* double knockout human cell line can be rescued by either *SCAF4* or *SCAF8*, supporting a common and essential function of these proteins. Based on that information, the client research study is working on collecting a cohort of individuals with *SCAF8* variants to better define this potential neurodevelopmental disorder.
  - **Role of GENYSIS:** Illustrates the benefits of a large research network and facilitating access to additional research.

### Continuous improvement and methodology developments

The UNC-CH PPMH has also supported a Research Specialist in the MGL who is responsible for the CLIA-certified Sanger sequencing and qPCR confirmation of GENYSIS research findings. Given that most client studies are not expected to have genomic sequencing performed in a CAP-accredited, CLIA-certified environment, we have relied on orthogonal confirmation in our clinical laboratory prior to clinical reporting. Historically, this has involved custom Sanger sequencing to confirm the variants identified in short-read sequencing. However, with incorporation of long-read sequence data and computational techniques to better recognize structural and copy-number variants, there is a continued need to validate techniques to confirm the presence of these variant types as well. We have utilized TaqMan quantitative Polymerase Chain Reaction (qPCR) analysis as this has proven to be a practical tool for confirming CNVs, offering targeted resolution without the added cost of genome-wide arrays. Sanger sequencing, while not suitable for CNV detection, has been valuable for confirming breakpoint-level changes in structural variants, especially in regions that are poorly resolved by short-read sequencing, but are too costly to validate using long-read sequencing technologies. We also leveraged qPCR-based expression analysis to assess the functional impact of regulatory elements and variants suspected to alter gene expression. In one case, transcript-level analysis provided evidence for reduced gene expression, supporting pathogenicity [21]. This integration of functional assays into the GENYSIS workflow has been especially useful for classifying variants. In other cases when Sanger sequencing and Taqman qPCR were not sufficient for confirmation, manually designed primers were used to selectively amplify the gene and not the pseudogene (*PMS2)*, allele-specific PCR was used to confirm phase (*SZT2)*, and long-read sequencing was utilized to sequence across breakpoints for a translocation/inversion (*ARID1B)*. When a clinically-validated confirmation test is not available through GENYSIS, a research report can be produced, providing recommendations to the research team about potential confirmatory testing to consider.

## Discussion

GENYSIS supports the advancement of genomic translational science and clinical genomic analysis in research studies by addressing several research barriers at the institutional and consortia level. Lack of transparency of results to participants has been a significant gap in genetics research [9,22–24]. By providing a clinical report of research results to both the patient and any of their providers who have access to the patient’s medical record, GENYSIS bridges the gap of utilizing genetic test results found during a research project for ongoing patient medical management. The GENYSIS core is also able to provide the analysis and reporting of secondary and incidental findings for studies that would otherwise not have had this capacity due to lack of analytic expertise or the resources to contextualize these results. These findings are not often included in research projects, especially those that lack the resources to provide post-test genetic counseling and follow-up recommendations for these results. GENYSIS has returned 6 secondary findings in the ACMG-recommended gene list and 3 other incidental findings to research participants. This included a secondary *RYR1* variant being identified before a scheduled sedated procedure so that precautions could be made regarding risk of malignant hyperthermia.

Another important benefit of GENYSIS is research participants obtaining access to additional research studies after their results have been returned. For example, participants with a novel gene variant reported by GENYSIS have been introduced to other research cohorts for further gene classification (cases 3, 4 and 5 in Box 2). One benefit of expert variant analysis, including use of GeneMatcher (a free database to enable connections between clinicians and researchers with common interest in particular genes [25]), is the enhanced ability to identify cohorts of patients with variants in the same gene as the participant. This allows enrichment of larger studies that may include ongoing functional studies to understand the mechanism of disease and potentially even develop therapeutic interventions.

It is also well understood that genetic research technologies can be more advanced with flexible pipelines compared to clinical laboratories. Pathogenic variants can be detected by advancements in sequencing and bioinformatic pipelines, such as those detected with long-read sequencing and/or structural variant analysis pipelines, and these can then be confirmed by more standard techniques such as Sanger sequencing or qPCR in a CLIA-CAP certified clinical laboratory. To meet the ongoing needs of the UNC clinical research community, GENYSIS is in continual improvement and development; for example, the group recently built a long-read sequence analysis pipeline for use with Oxford Nanopore Technology. Cases 1 and 2 from Box 2 demonstrate the ability to detect complex results in a research setting with a variety of emerging analytical technologies. The custom design for confirmatory testing is another benefit of the GENYSIS services.

Additionally, the GENYSIS core has worked with investigators to seek additional funding. We received an award to perform long-read sequencing on 10 prenatal genomes and pilot a pipeline for analysis of long-read sequencing data utilizing Oxford Nanopore Technologies’ EPI2ME [26]. The EPI2ME pipeline generates output files for single nucleotide polymorphisms, copy number variants, structural variants, repeat expansions, and methylation data that can be analyzed further with customized scripts and additional annotations and filtering.

Many lessons have been learned from the creation and work of the GENYSIS team:

### Provide modular services

Research projects vary in scope and aims, therefore a flexible workflow with a modular service menu that can be individualized for each project is essential. For example, post-test services are available, although optional; if the research team has a provider trained and available to return results, these services are unlikely to be needed. This also means the GENYSIS core team is responsible for knowing other resources that might be more appropriate for a project, if outside the scope of the core. This is often discovered during the first complementary consultation, as not all consultations have led to utilization of GENYSIS services. The group has often made recommendations for other services that may benefit the research project to a greater extent and our group aims to stay updated on the evolving resources available to our research community.

### Provision dedicated bioinformatics support

While the concept of bioinformatics as a service is not new, having it within the GENYSIS core allows our clients to focus on generating results without employing a full time bioinformatician of their own. This can be particularly helpful for smaller projects. As the number of groups working with genome sequencing increases, so do the number of tools and resources that are released. This creates an evolving environment with the need for consistent evaluation of tools needing to be replaced or updated. While in the past many UNC-CH genetic research projects have used their internally-developed pipelines, the GENYSIS team is now also implementing additional pipelines (e.g., EPI2ME, nf-core), which greatly reduces the workload on individual research projects.

### Systematize data management

With the amount of data being generated, the GENYSIS team needs regular processes to clean and remove unnecessary data to avoid reaching storage capacity. The team has discussions with individual projects to evaluate how long data is stored and later moving that data away from internal computing cluster, a process which can be complicated by university policies and procedures. These details are also important for projects to consider when writing their patient consent forms.

### Facilitate strategic sample collection

We had previously required duplicate patient samples so one could be exclusively used in the MGL for CLIA Sanger sequencing confirmation. We have greatly benefited from the BSP obtaining CAP accreditation, allowing direct use of this DNA extracted for CLIA Sanger confirmation. Additionally, the MGL is now validated to perform Sanger sequencing confirmation on DNA obtained from buccal swabs, as this sample collection methodology can reduce barriers for patients and families.

### Incorporate broad clinical and scientific expertise in genetic variant discussion

The consultation of various experts allows for a detailed presentation of variant data, while also considering the patient presentation, recorded phenotype, and relevant family history. This discussion often includes possible actionable treatment plans and post-reporting medical management with professionals firmly integrated within a variety of medical specialties. The Molecular Sign-out meeting is no longer held for a single project; multiple participant results from different projects are presented in one meeting. A confidentiality statement was created for all attendees, and all information is de-identified for confidential presentations.

### Include trainees

The bioinformatics knowledge corpus requires a deep understanding of biology, genetics, and computer science, with a strong emphasis on the willingness to constantly learn. The environment for this specific kind of education is unique and GENYSIS fosters the collaborative environment needed to learn these niche skills. Students are exposed to the procedures of IRBs and the intricate landscapes around human subjects’ data, including multi-layered discussions held at the Molecular Sign-out conferences. Students with clinical backgrounds are exposed to the complexities of research testing and variant curation, while bioinformatic students can transition from the world of statistics and python scripting to the complex world of genetic counseling. While most of the services provided by GENYSIS could be done without inclusion of trainees, their incorporation meets institutional goals to foster development of the next generation of genomic translational scientists. This core utilizes genomic medicine research projects as a venue for training future clinicians and researchers, contributing to the longevity and increasing capacity of this work for years to come.

### Garner institutional support

Support was particularly critical during the early phases of implementation. GENYSIS relied on departmental support as well as an ongoing collaboration with the PPMH. The PPMH provided foundational funding for the GENYSIS Core, covering core facility expenses and core facility personnel salaries during development. Since the GENYSIS Core became its own financial entity in July 2024, the PPMH has continued to support portions of GENYSIS personnel salary costs for research and development. As research core facilities are designed to operate on a breakeven model, the PPMH has also continued to provide end-of-year funding support for GENYSIS financial deficits. Finally, the PPMH has offered direct support for client studies piloting use of GENYSIS and an 80% subsidy on GENYSIS services for internal clients that are PPMH members, significantly reducing the cost of services. This includes the 17 academic institutions of the UNC System, offering wide access to GENYSIS services across the state of North Carolina.

We have many future goals. The GENYSIS team continues to work on standardized protocol language for consent forms to assist future projects when discussing genetics research with patients and to simplify review of future projects by IRBs through use of common text. We are developing training manuals and educational materials to aid in variant curation education. To date, we have only worked with researchers within our institution but hope to work with external clients, while recognizing that the additional complexity of incorporating a wider variety of input workflows for specimens or raw sequence data will require careful attention to quality control and sample tracking aspects.

## Conclusion

The UNC Clinical GENYSIS core facility implements analysis of genomic data and reporting of clinically relevant diagnostic or secondary findings. By offering integrated analytic and return-of-results capabilities, GENYSIS expands access to clinically actionable genomic findings for participants in research studies led by non-geneticists, thus advancing both translational genomic research and the ethical return of results in genomic research.
